# Age-Related Degree and Criteria Differences in Semantic Categorization

**DOI:** 10.5334/joc.74

**Published:** 2019-07-19

**Authors:** Steven Verheyen, Elisabeth Droeshout, Gert Storms

**Affiliations:** 1 Laboratory for Experimental Psychology, Faculty of Psychology and Educational Sciences, KU Leuven, Leuven, BE; 2 Laboratoire de Sciences Cognitives et Psycholinguistique, Département d’Études Cognitives, ENS, EHESS, PSL University, CNRS, Paris, FR

**Keywords:** Ageing, Categorisation, Mathematical modelling, Semantics

## Abstract

Individual differences in semantic categorization are commonplace. Individuals apply a word like SPORTS to different instances because they employ different conditions for category membership (vagueness in criteria) or because they differ regarding the extent to which they feel the term can be applied given fixed conditions (vagueness in degree). Three individuals may, for instance, disagree as to whether *chess* and *hiking* are SPORTS, because one believes SPORTS are competitive in nature, while the other two require SPORTS to be effortful (vagueness in criteria). On the basis of whether they consider *hiking* sufficiently effortful or not, the latter two individuals might still disagree as to whether to call it a SPORT (vagueness in degree). We investigated whether there are systematic age-related differences in semantic categorization by analyzing the categorization decisions of 1,868 adults for eight semantic categories with a formal model that allows the two sources of categorization differences to be disentangled. We found that young and older adults assess instances differently with respect to the categorization conditions and that older adults employ a lower threshold for category membership than young adults do. We recommend that these criteria and degree differences are taken into account in studies of age-related semantic processing.

## 1. Introduction

In semantic categorization tasks, participants indicate which candidate items they consider members of a target category. The current study is concerned with the question of whether semantic categorization shows age-related differences and if so, what the nature of those differences is. Because of pronounced individual differences in the application of common language terms, this question is not straightforward to answer. Most language terms are vague and vague terms can be used in different manners without being erroneous (Kölbel, 2004; Raffman, 2014; Wright, 1995). That is, even when obvious sources of individual differences – such as the context of an utterance or the communicative goal of the speaker – are controlled for, there remain considerable individual differences in the manner in which terms are applied, with individuals diverging widely about the instances they consider words as FURNITURE or SPORTS to apply to (Black, 1937; Borel, 1907; McCloskey & Glucksberg, 1978; Verheyen, Hampton, & Storms, 2010). The natural occurrence of these individual differences needs to be taken into account in any study of age-related meaning differences of these terms (Verheyen, Ameel, & Storms, 2011; White, Storms, Malt, & Verheyen, 2018). That is, in order to investigate what differs between age groups (if anything), an account of the nature of these individual differences is required.

The vagueness literature indicates what types of individual differences one should account for in categorization studies. Two sources of individual differences are generally recognized: degree differences and criteria differences (Alston, 1964; Burks, 1946; Devos, 1995, 2003; Kennedy, 2013; Machina, 1976; Verheyen and Storms, 2018). We will follow Devos (1995, 2003) in defining vagueness in criteria (sometimes referred to as intensional vagueness) as the indeterminacy with respect to (the combination of) the conditions for application of a term. When two individuals disagree as to whether *video game tester* or *homemaker* should be considered a PROFESSION because the first individual requires PROFESSIONS to provide an income, while the second requires them to be effortful, vagueness in criteria is in play. Devos defines vagueness in degree (also referred to as extensional vagueness) as the extent to which a term can be applied given that the conditions have been determined. That is, even when two individuals agree that for something to be considered a PROFESSION, it should require effort, they might disagree as to whether *video game testing* is sufficiently effortful to be considered a PROFESSION.

The current study aims to determine whether there are age-related degree and criteria differences by analyzing the semantic categorization data of young and older adults with a formal model that allows the two sources of individual differences to be disentangled. Although, to our knowledge, this qualification of differences has not been made explicit in the aging literature, a number of studies can be interpreted as providing evidence for both criteria and degree differences between young and older adults. We discuss these studies in the next sections before we turn to an exposition of the modeling framework (section 2) and the empirical study we conducted (section 3).

### 1.1. Age-related degree differences in categorization

The metaphor of the mental lexicon as a network is a recurring one ever since the work of Collins and colleagues (Collins & Loftus, 1975; Collins & Quillian, 1969). The meaning of a node in the network, corresponding to a word, is found in its connections with other nodes in the network. As a word is heard or read, its node becomes activated and this activation spreads to other nodes to which it is connected, thus giving rise to a distributed representation of its meaning. The metaphor is also commonly found in theories in the aging literature. Several of these theories give rise to the hypothesis of an age-related degree difference in categorization, whereby older adults entertain broader categories than younger adults do because they do not require instances to meet the category membership criteria to the same degree as younger adults.

The aging literature harbors at least two reasons for expecting older adults to include more exemplars in their categories than younger adults do. The first is a higher resting level of activation for nodes in the older adults’ networks because older adults have more extensive experience with the corresponding concepts (Buchler & Reder, 2007). As a result, in a semantic categorization task nodes in the older adult’s network would require fewer impulses to turn on in response to the presentation of a target category. The second reason pertains to a more elaborate spread of activation through the older adults’ network. That is, in semantic categorization tasks the presentation of a target category to older adults would activate nodes that are not reached in the younger adults’ networks. This may be due to older adults taking more time to respond and thus allowing activation to spread longer compared to young adults (Laver & Burke, 1993) and/or older adults having a denser network due to more diverse experience with the concepts that constitute the network (Buchler & Reder, 2007). This second reason is sometimes worded more negatively as the inhibitory deficit hypothesis, whereby older adults are supposedly less able to suppress activated information that is only remotely related to the target category or the task at hand, resulting in the endorsement of very atypical exemplars or even loose associates of the category (Hasher et al. 1999; see also Carlson et al. 1995).

Evidence in favor of more elaborate semantic networks in older age comes from semantic representations built from word co-occurrences in text corpora elicited from young and older adults. The representations of the latter are characterized by denser word neighborhoods (Conley & Burgess, 2000a, 2000b). In word association studies, the interconnections between words are elicited in a direct manner, by having participants answer with the first words that come to mind in response to a cue word. These studies provide mixed support for more elaborate semantic networks in older age. Some studies have indeed shown more variable word associations in older age (Dubossarsky, De Deyne, & Hills, 2017; Tresselt & Mayznet, 1964), but the effect appears to diminish when vocabulary level is controlled for (Burke & Peters, 1986; Lovelace & Cooley, 1982; Scialfa & Margolis, 1986). One study found the opposite effect in that older participants demonstrated less response heterogeneity (Hirsh & Tree, 2001), while yet another study found the network structure of young and older adults to be similar (Zortea, Menegola, Villavicencio, & Salles, 2014).

### 1.2. Age-related criteria differences in categorization

While the case for age-related degree differences in categorization was made both on theoretical and empirical grounds, the case for criteria differences is made primarily on empirical grounds. As was the case for the degree difference, the empirical evidence in favor of age-related criteria differences is mostly indirect, but does suggest that there are categories for which young and older adults diverge regarding the criteria they deem important for membership.

In the category fluency task participants are invited to list as many exemplars of a target category as they can in the span of a minute. Participants tend to start off with the exemplars they consider the best examples of the category, before moving on to more atypical ones (Hampton & Gardiner, 1983; Mervis, Catlin, & Rosch, 1976). To the extent that the order of the generated exemplars is different in young and older adults, the category fluency data suggest that they employ different criteria for category membership (Brosseau & Cohen, 1996). The responses produced by young and older participants in the word association task differ markedly as well, again suggesting a difference in the information content that is emphasized in both groups (Hirsh & Tree, 2001).

More direct evidence for age-related criteria differences comes from a study by Howard (1983) in which participants were to judge the similarity of several animals. While the oldest participants tended to base their judgments on the animals’ size, the middle-aged participants emphasized the animals’ predativity. In a study about the use of container names such as BOTTLES and BOXES, White, Storms, Malt, and Verheyen (2018) found older adults to emphasize materials such as glass or cardboard in their category membership decisions, whereas younger adults emphasized more “modern” materials such as plastics.

The use of different categorization criteria by younger and older participants can also show at the level of individual target instances, when they are not awarded the same membership status by participants of different ages. While younger adults assert that both *dial phones* and *cell phones* are ‘really’ PHONES, older adults only consider the former ‘really’ category exemplars, for instance (Malt & Paquet, 2013). A similar finding is presented in a study by Little, Prentice, and Wingfield (2004) on young and older adults’ sensitivity to the goodness of fit of words in the contexts of meaningful sentences. Individual items were assessed differently by young and older adults. An illustrative example is observed for the sentence frame “*When the music played, she remembered her first —— lesson*.” Whereas both age groups ranked *ballet* highest (young = 6.55, older = 7.67), the young adults gave *salsa* a mean rating of 4.91, while the older adults gave it a mean rating of only 1.40. In the same sentence, *polka* had the second highest rating for the older adults (7.50), whereas it was rated seventh highest by the young adults (5.00). Dissociations like these indicate that the conditions of application of category terms can be different in the two groups.

The idea that young and older participants’ beliefs about individual exemplars may differ is also found in Pennequin, Fontaine, Bonthoux, Scheuner, and Blaye (2006). Both in free sorting and match to sample tasks, taxonomic classifications have been shown to decrease with age, while thematic classifications tend to increase (e.g., Annett, 1959; Cicirelli, 1976; Smiley & Brown, 1979). Pennequin et al. attribute these classification differences to differences in the assessment of exemplars’ associative strength by young and older adults (see also Kogan, 1974, for a related argument). They propose that the strength of the associative relationship between an exemplar and a target category may differ between age groups because associative strength depends on experience, and the experiences of young and older adults may differ. Accordingly, when they took age differences in the judgments of associative strength into account, age differences in classification no longer showed.

### 1.3. Aim

The direct motivation for this paper was a study on semantic memory in healthy old age by Morrow and Duffy (2005). They asked participants to rate the typicality of instances for different categories on a 7-point Likert scale. Morrow and Duffy found that the average ratings of young and older participants correlated strongly (with correlations ranging between .701 and .929; *M* = .861 across 12 semantic categories), but that the ratings from the older adults (+62-year-olds) were significantly higher (in all but 2 categories). The former finding indicates that both cohorts tend to agree on the relative ordering of instances in terms of category representativeness, and thus suggests that young and older adults employ similar criteria. However, when the variability within each group is taken into account and these correlations are corrected for attenuation, they fall short of indicating perfect agreement between the age groups, which could be due to age-related criteria-differences. The finding of higher ratings by the older participants could be taken to indicate the degree difference hypothesized above, whereby older adults are less conservative when it comes to category membership, but it can also be due to a different use of the Likert scale by the older participants (i.e., they may consistently use higher scale values). In this paper we will investigate how the observations by Morrow and Duffy can be interpreted in terms of age-related criteria and degree differences.

Based on the evidence reviewed in sections 1.1 and 1.2, there seem to be sufficient grounds to raise the hypothesis of an age-related degree difference in semantic categorization, whereby older adults entertain broader categories than young adults do. While the proposed degree difference is thought to hold across categories, it is to be expected that whether criteria-related differences appear will be dependent upon the category under investigation. For instance, the fact that age-related criteria differences were found in White et al.’s (2018) study of container categories can be explained by the fact that the manufacturing process of the artefacts under study underwent a change in the lifetime of the older participants (i.e., increased use of plastics). Similarly, the different conception of a category like PHONE in Malt and Paquet (2013) is most likely the result of a shift in the primary application of the targeted category. Explanations like these do not hold across the entire conceptual domain, but only apply to particular categories.

The following section introduces a formal model that allows one to characterize group differences in categorization as either degree or criteria differences. The model is a general-purpose one in that it can be applied to any categorization task that pertains to vague terms (and thus yields many individual differences) for which one wants to look at group differences (be it participants in different conditions or with a different background). We will apply it here to semantic categorization data from nearly 2,000 young and older adults to test the hypothesized age-related degree and criteria differences.

## 2. Theoretical Framework

To analyze the semantic categorization data, we will employ a statistical model that was originally introduced in the psychometric literature to detect group differences and bias in high stakes testing situations. The Random Item Mixture Model (RIM; Frederickx, Tuerlinckx, De Boeck, & Magis, 2010) is generally applied to individuals’ responses to test items in order to simultaneously infer the items’ difficulty and the individuals’ ability with respect to the test construct. In this context, a group difference emerges when the average ability of one group of test takers is reliably different from the average ability of another group of test takers. Bias is identified when test takers with the same ability, who belong to different groups, have a different probability of answering the same test item correctly. This might occur when the item, for instance, presupposes cultural knowledge, which one of the groups does not possess. The item is then said to function differently in the two groups (Embretson & Reise, 2000).

In a similar vein, the RIM model can be applied to participants’ semantic categorization responses to detect degree and criteria differences between groups of categorizers (Stukken, Verheyen, & Storms, 2013; Verheyen & Storms, 2018). On a test, a test taker’s high ability will manifest itself in a large number of correct responses, while an item’s difficulty will manifest itself in the number of test takers that get the item correct (fewer correct responses indicating a more difficult item). The test situation is analogous to that of the semantic categorization task, in which the endorsement of many items as category members indicates that the categorizer is lenient rather than conservative, and an item that is regularly endorsed as a category member can be considered to meet the categorization criteria well (Verheyen, Hampton, & Storms, 2010). A degree difference between two groups of categorizers would then show in the average leniency difference between the groups, while criteria differences would show as items functioning differently in the groups. The probability of endorsing an item as a category member should be the same for two categorizers from distinct groups who are matched in terms of leniency, IF they were to use the same categorization criteria. Violations would indicate that they do not.

The RIM model can be cast in the terminology of the Threshold Theory (Hampton, 1998, 2007), a theoretical framework put forward to explain individual differences in semantic categorization by appealing to vagueness in criteria (Hampton, 2006) and degree (Hampton, 1995). Categorization decisions are regarded the outcomes of a probabilistic decision process that operates on a latent dimension (Verheyen, Hampton, & Storms, 2010). The latent dimension can comprise one (Verheyen, Dewil, & Egré, 2018) or a weighted combination of several (Verheyen, De Deyne, Dry, & Storms, 2011) substantive criteria. The items’ positions on the latent dimension reflect the extent to which they meet the categorization criteria, with items being positioned further down the dimension, the more they fulfill the categorization criteria. One value along the latent dimension corresponds to the point of subjective equality and reflects the degree of the categorization criterion for which one feels equally inclined to apply and to deny the category label. The categorization of individual items depends on their relative position and distance to this point, with the likelihood of a member response increasing the more an item surpasses it, and the likelihood of a non-member response increasing the more an item falls short of it (Verheyen, Hampton, & Storms, 2010). This tipping point is generally referred to as a threshold to indicate that it reflects the degree of the categorization criterion that warrants a positive rather than a negative categorization decision (Hampton, 1995, 1998, 2007). It expresses categorizers’ leniency in that the category extension decreases the further down the dimension this threshold is positioned, indicating that the category membership requirements increase. Resuming the PROFESSIONS example from the introduction, the latent dimension could reflect effortfulness, with activities positioned further down the dimension the higher the level of exertion involved. A categorizer’s threshold would then indicate the level of exertion required for activities to be considered PROFESSIONS.

Formally, the probability that categorizer *c* decides that item *i* is a category member (Y_ci_ = 1) as opposed to a non-member (Y_ci_ = 0) is a logistic function of the distance between the position of the categorizer’s threshold *θ_c_* and the position of the item *β_i_* along the latent dimension:

{\rm{Pr}}\left({{{\rm{Y}}_{ci}}=1}\right)=\frac{{{e^{{\beta_i}-{\theta_c}}}}}{{1+{e^{{\beta_i}-{\theta_c}}}}}

Both the item and the threshold positions are estimated from the semantic categorization data based on the relative frequency with which items are categorized and participants endorse items as category members.

In Threshold Theory terms, vagueness in degree in this model shows in the different thresholds the categorizers employ (Stukken, Verheyen, & Storms, 2013; Verheyen & Storms, 2018). Whether items are regarded category members or not, will depend on their relative position to the categorizers’ thresholds. Few items will exceed a conservative categorizer’s threshold that is positioned on the far right side of the latent dimension, while many items will exceed a lenient categorizer’s threshold positioned on the far left side of the dimension. Individual differences in category extension can thus come about through the use of different thresholds. Categorizers with different thresholds effectively require items to meet different degrees of the categorization criterion for category membership (e.g., different levels of exertion for PROFESSIONS). Differences in severity between groups can then be explored by comparing the mean threshold estimates of the groups (see Verheyen, Ameel, & Storms, 2011, for an example). To this end, every threshold parameter *θ_c_* in the RIM model is supplemented with an index *g* indicating the group categorizer *c* belongs to:

{\rm Pr}\left({\rm Y}_{cig} = 1 \right) = \frac{e^{{\beta_\iota} - \theta_{cg}}} {1 + e^{{\beta_\iota} - \theta _{cg}}}

Vagueness in criteria shows in differences in item positions (Stukken, Verheyen, & Storms, 2013; Verheyen & Storms, 2018). In the context of semantic categorization, an item functions differently when participants demonstrate a different probability of endorsing an item despite employing the same threshold. With identical thresholds, this can only come about in the model if the item is positioned differently, since the endorsement probability is determined by the relative distance of the item from the threshold. Seeing that the items’ positions on the latent dimension reflect the extent to which they meet the categorization criteria, differently positioned items indicate that there are criteria differences. Criteria differences between groups of categorizers can be subtle, affecting the position of a limited number of items (Stukken, Verheyen, & Storms, 2013; Verheyen & Storms, 2018), or can apply to the entire set of items, yielding a significant reorganization of the latent dimension (Verheyen & Storms, 2013; Verheyen, Voorspoels, & Storms, 2015). While the latter case indicates the use of clearly distinguishable criteria, the former case suggests the affected items are assessed differently with respect to the same categorization criterion by the members of the two groups or – when the latent dimension is a composite of several criteria – that these criteria were weighted differently in the two groups. Consider the PROFESSIONS example again, where the effortfulness of a particular activity like *homemaker* or *video game tester* could be differently assessed in two groups of categorizers or where activities could be positioned according to their level of exertion in one group vs. the income they provide in another group. While the latter case is a clear example of the use of different substantive criteria, in the former case the two groups employ the same substantive criterion (effortfulness) yet their judgment of the effortfulness of individual exemplars differs (one group may find that *video game testing* requires a lot of effort, while the other group believes it easy). This should not be mistaken for a degree difference in that they both may impose the same threshold level of exertion for activities to be considered PROFESSIONS. If that threshold level happened to be high, the fact that *video game tester* is considered a PROFESSION in the first group, but not in the second, is not due to a different positioning of the categorization threshold, but the result of a different positioning of the item. We consider all these cases demonstrations of vagueness in criteria since they all pertain to differences in the latent dimension on which the categorization process operates.^1^

The way in which the RIM model allows criteria differences between groups to be explored is through the introduction of a latent indicator C_*i*_ which indicates whether item *i* does (1) or does not (0) function differently in the two groups of categorizers. Items that do have a different probability of being endorsed by threshold-matched members of distinct groups, warrant the inclusion of separate item positions indicated by an index *g*:

{\rm{Pr}}\left({{{\rm{Y}}_{cig}} = 1|{{\rm{C}}_i} = 1} \right) = \frac{{{e^{{\beta_{ig}} - {\theta _{cg}}}}}}{{1 + {e^{{\beta _{ig}} - {\theta _{cg}}}}}},

while items that function the same, do not:

{\rm{Pr}}\left({{{\rm{Y}}_{cig}} = 1|{{\rm{C}}_i} = 0} \right) = \frac{{{e^{{\beta_i} - {\theta _{cg}}}}}}{{1 + {e^{{\beta _i} - {\theta _{cg}}}}}}.

While the position of the former items is dependent upon the group (indicated by the addition of index *g* to *β_i_*), the position of the latter items is not (*β_i_* does not receive an index *g*).

## 3. Method

### 3.1. Participants

1,877 individuals from across Flanders (Belgium) participated in a web survey. They learned about the survey through a flyer, personal communication, e-mail, or social media. To ensure that older participants were well represented in the participant sample, we approached seniors organizations and centers for adult education. The data of nine participants who were not of adult age (<18 years old) were omitted.

The native language of the remaining 1,868 participants was Dutch. 1,036 participants (55%) identified as female. The others identified as male. A small percentage of participants never obtained a diploma (1%). For 4% the highest diploma obtained was that of primary education; for 31% it was that of secondary education. The remainder of the participants obtained a diploma beyond the compulsory level, either at a university college through a short (27%) or a long program (16%) or at a university (17%). Three percent of the participants went on to obtain a PhD. Following Morrow and Duffy (2005) we identified participants younger than 62 years old as young adults (42%) and participants aged 62 and older as older adults (58%).^2^ Table 1 provides an overview of the number of participants per combination of age group, gender, and education level.

**Table 1:** Distribution of the number of participants according to age group (young vs older adults), gender (males vs females), and education level (highest diploma obtained).

Age group	Highest diploma obtained						

	No diploma	Primary education	Secondary education	University college (short)	University college (long)	University	PhD

**Males**							

Young	2	5	75	43	24	48	15
Older	7	19	196	140	114	124	20
**Females**							

Young	6	15	168	165	91	116	11
Older	1	33	147	165	78	38	2

We separated the data from male and female participants, because semantic categorization is known to be affected by gender (Kempton, 1981; Stukken, Verheyen, & Storms, 2013; Verheyen, Dewil, & Egré, 2018). Among the male participants, age ranged from 18–61 years (*M* = 48.9, *SD* = 14.2) in the younger group and from 62–91 years (*M* = 70.00, *SD* = 5.80) in the older group. Among the female participants, age ranged from 18–61 years (*M* = 46.00, *SD* = 15.70) in the younger group and from 62–92 years (*M* = 68.20, *SD* = 5.20) in the older group.

Because of a significant relationship between education level and age group for the female participants (the younger women tended to be more highly educated; χ^2^(6, *N* = 1036) = 47.72, *p* < .001), an equated sample was constructed. For each education level, the age group with the smallest number of participants was determined and the data from these *n* participants were combined with the data of the first *n* participants of the same education level from the other age group. The equated sample thus includes for each education level an equal number of young and older female participants. By design χ^2^= 0 with *p* = 1 in this equated sample since young and older female participants are evenly distributed across the education levels. Among male participants the relationship between education level and age group was not significant (χ^2^(6, *N* = 832) = 12.24, *p* = .06).

Out of all participants, 1,773 (95%) completed the web survey. The remaining 95 respondents (5%) partially filled out the web survey, answering at least one semantic category fully. As a result, the final number of participants differed slightly from category to category. For males there were at least 199 young and 600 older participants and for females at least 512 young and 489 older. When equated for education the female numbers were at least 433 in each category.

### 3.2. Materials

Eight categories, with 24 items each, were taken from Verheyen, Hampton, and Storms (2010). They were Dutch translations of the materials from Hampton, Dubois, and Yeh (2006). The eight categories comprise animal categories (FISH and INSECTS), artifact categories (FURNITURE and TOOLS), borderline artifact-natural kind categories (FRUIT and VEGETABLES) and activity categories (SCIENCES and SPORTS). The category items comprise several clear members and clear non-members, but mainly borderline cases, for which individual differences in opinions about category membership were expected. For the category of SPORTS, for example, the items included clear members such as *tennis* and *swimming*, clear non-members such as *picnicking* and *talking*, and a variety of borderline cases such as *billiards, bullfighting, chess, darts, hiking, hunting*, and *kite flying* (among others). The items *lettuce* and *spinach* are clear members of the category VEGETABLES, while *apple* and *pineapple* clearly do not belong to the category. Examples of borderline cases for the category VEGETABLES are *bamboo shoot, garlic, parsley, potato, sage, seaweed*, and *soybean*. See Appendix A for a full list of the materials.

### 3.3. Procedure

Participants completed a web survey that comprised the informed consent, demographics (gender, age, native language, highest diploma obtained), and the semantic categorization task. For each target category, the participants were asked to decide whether the items were category members or not. They could also indicate that they did not know a particular item. The presentation order of both the categories and the items within a category was randomized for every new participant. It was emphasized that participants’ personal opinions mattered, and the use of web search engines or other reference materials was discouraged. Participants could proceed at their own pace. The majority of the participants completed the survey in less than ten minutes.

The semantic categorization task was untimed so as not to confound categorization differences with reaction time differences (Phillips, 1999; see also Giffard, Desgranges, Kerrouche, Piolino, & Eustache, 2003; Laver, 2009; Laver & Burke, 1993; Myerson, Hale, Chen, & Lawrence, 1997, on cognitive slowing). Unlike the tasks that have been reviewed in the introduction, the use of a semantic categorization task provides a more direct measure of the category extension participants entertain. Because of its binary nature, it is less susceptible to individual difference in scale use than, for instance, the Likert scales used for typicality ratings. Finally, the semantic categorization task depends less on episodic memory than recall tasks like exemplar generation or word association do, which can be strenuous for older adults (Light, 2000).^3^

### 3.4. Model analysis

We used the RIM model to analyze the semantic categorization data for indications of degree and criteria differences between young and older adults. In what follows we will index model parameters pertaining to young adults with *Y* and model parameters pertaining to older adults with *O*. Since we wanted to allow the possibility that the threshold distributions of young and older adults differ, we assumed the thresholds *θ_c_* to follow a normal distribution with a group-specific mean *µ* and variance *σ*^2^:

\begin{array}{*{20}{l}}
{{\theta _{cY}} \sim N\left({{\mu _{{\theta _Y}}}, \sigma _{{\theta _Y}}^2} \right)}&{{\rm{for\ the\ young\ adults,\ and}}}\\
{{\theta _{cO}} \sim N\left({{\mu _{{\theta _O}}}, \sigma _{{\theta _O}}^2} \right)}&{{\rm{for\ the\ older\ adults}}}.
\end{array}

Because the RIM model assumes that the probability that categorizer *c* endorses item *i* as a category member depends on the *distance* between the position of the categorizer’s threshold *θ_c_* and the position of the item *β_i_*, the model is underidentified. Adding any constant to both *θ_c_* and *β_i_* will leave the likelihood unchanged. To identify the model, the mean threshold value for the young group {\mu _{{\theta _Y}}} was set to 0. As a result, the mean threshold value for the older group {\mu _{{\theta _O}}} can be thought of as the mean threshold difference between the groups.

We consider the items in this study to constitute a random sample of the population of items that could potentially be presented for categorization. We did not sample them in a systematic fashion and have no particular interest in how this specific set of items is categorized by young and older participants. Rather, we consider them representative of a broader population of items. We therefore assume the item positions to follow a normal distribution with common mean:

{\beta _i}|{C_i} = 0\sim N\left( {{\mu _\beta },\,\,\sigma _\beta ^2} \right) for items functioning similarly in younger and older adults, and

\left. {\left({\begin{array}{*{20}{c}}
{{\beta _{iY}}}\\
{{\beta _{iO}}}
\end{array}} \right)\,} \right|{C_i} = 1\sim N\left[ {\left({\begin{array}{*{20}{c}}
{{\mu _\beta }}\\
{{\mu _\beta }}
\end{array}} \right),\,\,\left({\begin{array}{*{20}{c}}
{\sigma _{{\beta _Y}}^2}\\
{{\sigma _{{\beta _Y}{\beta _O}}}}
\end{array}\begin{array}{*{20}{c}}
{{\sigma _{{\beta _Y}{\beta _O}}}}\\
{\sigma _{{\beta _O}}^2}
\end{array}} \right)} \right] for items functioning differently in the two groups.

In the former case the item positions follow a univariate distribution, meaning that they are the same for young and older categorizers (*β_i_* has no group index). In the latter case they are drawn from a bivariate distribution, meaning that the item positions are different for the young and older categorizers (there is a separate *β_iY_* and *β_iO_* for the younger and older group, respectively).

The classification of items that do and do not function differently in the young and the older group was achieved using Bernoulli distributed latent indicators *C_i_*:

{C_i} \sim Bern\left(\pi \right).

The RIM model parameters were inferred using the WinBUGS software for Bayesian model estimation using Markov chain Monte Carlo methods (Lunn, Thomas, Best, & Spiegelhalter, 2000) according to the procedure outlined by Frederickx et al. (2010). These included the specification of standard normal priors for the mean parameters *μβ* and {\mu _{{\theta _O}}}, uniform priors between 0 and 3 for the standard deviations {\sigma _{{\theta _Y}}}, {\sigma _{{\theta _O}}}, {\sigma _{{\beta _Y}}}, and {\sigma _{{\beta _O}}}, and a Beta prior with both shape parameters set to 1 for *π*, as well as the estimation of the correlation rather than the covariance parameter. A uniform distribution between –1 and 1 was set as prior for this correlation.

For every category separate analyses were run on the semantic categorization data of the male, female, and education equated female participants. Each of these 8 × 3 analyses involved five chains of 10,000 iterations each, with a burn-in sample of 1,000.

## 4. Results

We assessed the convergence of the chains with the \hat R criterion, which is approximately the square root of the ratio of the between-chain variance to the within-chain variance (Brooks & Gelman, 1998). It is recommended that \hat R \le 1.1 for all parameters, although a criterion value of 1.5 is also acceptable when sampling proceeds slowly (Gelman & Hill, 2007). Across analyses, \hat R was smaller than 1.1 for 99.74% of the parameters. When \hat R exceeded 1.1, it was always smaller than 1.5 indicating that there were no parameters with extremely poor convergence.

In what follows, we will start by reporting the evidence for degree differences in semantic categorization between young and older adults, before turning to criteria differences. A third subsection is devoted to the differences in categorization patterns as a whole that emerge by combining the degree and criteria differences.

### 4.1. Degree differences

We hypothesized that older adults would use a lower threshold compared to young adults across categories. Since the mean threshold value for the young group {\mu _{{\theta _Y}}} was set to 0 (see section 3.4 Model analysis), the mean threshold value for the older group {\mu _{{\theta _O}}} can be thought of as the mean threshold difference between the groups. It can be used to determine whether the two age groups differ regarding the degree they feel the target categories apply. A difference in degree is deemed reliable if the 95% credibility interval for {\mu _{{\theta _O}}} does not include 0. Table 2 holds the lower and upper boundaries of the 95% credibility intervals of {\mu _{{\theta _O}}} for every category, along with the median of the posterior distribution. Reliable group differences are set in bold.

**Table 2:** Posterior distribution of the mean threshold difference between young and older adults, separated for Male, Female, and Female data that were Equated for Education level. Reliable group differences are set in bold.

Category	Male			Female			Female Education Equated		

	Pct 2.5	Pct 50	Pct 97.5	Pct 2.5	Pct 50	Pct 97.5	Pct 2.5	Pct 50	Pct 97.5

FISH	**–0.66**	**–0.34**	**–0.06**	**–0.70**	**–0.44**	**–0.17**	**–0.57**	**–0.28**	**–0.01**
INSECTS	–0.30	–0.09	0.14	–0.09	0.16	0.37	–0.13	0.13	0.35
FURNITURE	–0.60	–0.28	0.02	–0.41	–0.06	0.25	–0.27	0.06	0.41
TOOLS	–0.53	–0.26	0.06	**–0.58**	**–0.35**	**–0.08**	–0.61	–0.31	0.03
FRUIT	–0.43	–0.14	0.10	**–0.56**	**–0.37**	**–0.13**	–0.52	–0.21	0.01
VEGETABLES	**–0.66**	**–0.42**	**–0.17**	**–0.63**	**–0.46**	**–0.28**	**–0.62**	**–0.45**	**–0.27**
SCIENCES	–0.28	–0.02	0.26	–0.14	0.06	0.26	–0.28	–0.06	0.15
SPORTS	–0.22	0.02	0.23	–0.32	–0.13	0.06	–0.33	–0.14	0.06

Across categories and data sets, the median of the posterior distribution of {\mu _{{\theta _O}}} was negative (except for SPORTS in the Male data, INSECTS and SCIENCES in the Female data, and INSECTS and FURNITURE in the Female Education Equated data). The negative values for {\mu _{{\theta _O}}} indicate that the average threshold for older adults is lower (located more to the left of the categorization dimension) than the average threshold for young adults is. At older age, people thus tend to be less severe in their semantic categorization decisions. However, only in the case of FISH and VEGETABLES (in both the Male, Female, and Female Education Equated data) and TOOLS and FRUIT (in the Female data), were these threshold differences reliable and can we confidently say that older participants do not require items to meet the categorization criteria to the same degree as young participants. The observation that the median of the posterior distribution of {\mu _{{\theta _O}}} was negative 19 out of 24 times (79%) is nevertheless indicative of a general degree difference between young and older adults. If participants were randomly assigned to groups (irrespective of their age) we would only expect a negative threshold difference half of the time (see Appendix B for a Monte Carlo simulation study in support of this claim).

The analysis of the data of all the female participants yields reliable threshold differences for the categories TOOLS and FRUIT, in addition to VEGETABLES and FISH. That is, without controlling for education level, degree differences are observed for more categories. The young female adults were more highly educated compared to the older female adults (see section 3.1 Participants). This suggests an effect of education level on semantic categorization decisions, whereby more highly educated individuals are more strict when it comes to semantic categorization (see Verheyen & Storms, 2018, for an extensive treatment of education differences in semantic categorization).^4^

### 4.2. Criteria differences

We hypothesized that criteria differences between young and older adults would be category-dependent. Not all categories would necessarily show an age-related criteria difference since not all items are subject to age-related experience differences. In the RIM model, item indicators C_*i*_ indicate whether items function differently in the young and the older age groups (i.e., have a different probability of being endorsed despite matched thresholds). Items are classified as functioning differently when the posterior probability of indicator C_*i*_ exceeds .50. Since this indicates that the item is positioned differently in the two groups (see section 3.4 Model analysis), it also signals that young and older participants employ different criteria for semantic categorization. Table 3 gives an overview of the number of items for each category that were identified as functioning differently. The stated numbers indicate how many of these items were relatively more often endorsed by young and by older adults. (See Appendix A for an overview of the differently functioning items.)

**Table 3:** Number of items functioning differently in young and older adults for the Male, Female, and Female Education Equated data, divided according to whether they are more often endorsed by the young or the older adults.

Category	Male		Female		Female Education Equated	

	Young	Older	Young	Older	Young	Older

FISH	1	1	2	1	2	2
INSECTS	1	2	2	5	2	4
FURNITURE	1	1	4	2	2	2
TOOLS	2	1	0	3	1	3
FRUIT	2	3	2	1	1	4
VEGETABLES	0	1	1	1	1	0
SCIENCES	0	1	0	0	0	0
SPORTS	0	1	2	0	2	0
Total	7	11	13	13	11	15

Across data sets, an average of 2.92 items per category (out of 24) were identified as functioning differently in the young and older adults. This is substantially more than one would expect if there were no systematic age differences between the groups. Monte Carlo simulations indicate that if young and older participants are randomly assigned to groups, only 0.02 items per category are expected to function differently (see Appendix B). Only for the category SCIENCES in the Female and Female Education Equated data, did we not identify at least one differently functioning item.

Despite the fact that several items functioned differently in the two age groups, high correlations were observed between the posterior means of the item positions *β_i_* in the young and older groups for all of the categories. Both in the Male, Female, and Female Education Equated group, the correlations were higher than 0.99. That is, despite some items being positioned differently along the categorization dimensions of the two groups, the relative order of items was retained. This indicates that the observed criteria differences pertain to individual items rather than the categories as a whole.

The items that were identified as functioning differently in the two age groups were not systematically more probable to be endorsed by the older than by the younger adults (or vice versa).^5^ Nor did this seem to be systematically affected by gender or category. Within each participant group, one can find categories where the number of differently functioning items that are more probably to be endorsed by the older than by the young adults is higher, the same, or lower (see, for instance, the categories INSECTS, FURNITURE, and TOOLS, in the Male participant group). For the category VEGETABLES, the number of differently functioning items that were more probable to be endorsed by the older adults is higher (Male data), the same (Female data), or lower (Female Education Equated data) depending on the participant group.

There was no clear effect of education level. Comparing the Female Education Equated data with the entire Female data set, we see that the number of differently functioning items can increase or decrease, depending on the category.

### 4.3. Categorization patterns

Through the model analyses, we identified categories (i) without degree and criteria differences, (ii) with criteria but no degree differences, and (iii) with both degree and criteria differences. Neither for the male, female, nor female education equated group did we identify a category with degree but no criteria differences. To aid the understanding of what these patterns mean for the categorization behavior of the young and older adults, we provide figures depicting the proportion of participants in the young (black circles) and older group (gray squares) endorsing items as category members.

**Figure 1:**
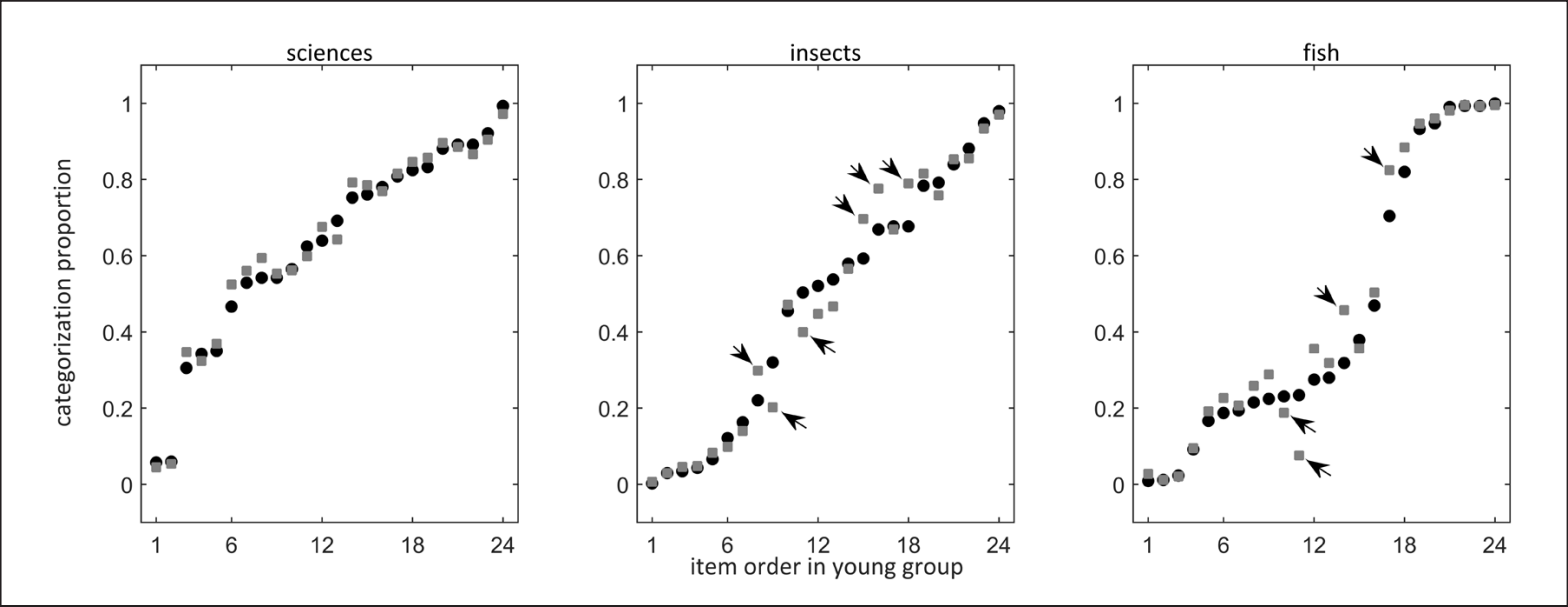
Categorization proportions for SCIENCES (left), INSECTS (middle), and FISH (right) of the young (black circles) and the older adults (gray squares) in the Female Education Equated group. Items are ordered along the horizontal axis according to their categorization rank in the young group. Arrows indicate items that function differently in the young and older adults.

We employ the Female Education Equated data for illustratory purposes. The left panel of Figure 1 contains the categorization proportions for SCIENCES, the only category without degree and criteria differences. The middle panel of Figure 1 contains the categorization proportions for INSECTS, one of the categories without a degree, but with criteria differences. The right panel of Figure 1 contains the categorization proportions for FISH, one of the categories with both degree and criteria differences. The items are organized along the horizontal axis in increasing order of endorsement according to the young group. The item on the far left is thus the one least endorsed by the young participants (black circles), while the item on the right is the one most endorsed by these participants.

The category SCIENCES was the only one in the Female Education Equated group without a credible degree difference (see Table 2) or differently functioning items (see Table 3). In the left panel of Figure 1 this shows in the absence of marked differences between the categorization proportions of the young and older adults.

For the categories INSECTS, FURNITURE, TOOLS, SPORTS, and FRUIT, we observed no credible degree difference, but we did find items functioning differently in the young and older group. In the middle panel of Figure 1, we see for the category of INSECTS that these are indeed the items for which the categorization proportions of the young and older participants differ the most (indicated by arrows). Items 8, 15, 16, and 18 (corresponding to *scorpion, louse, spider*, and *mite*) were more endorsed by older adults, while items 9 and 11 (corresponding to *worm* and *maggot*) were more endorsed by young adults.

For the category FISH, a credible degree difference indicated that older adults employ a lower threshold than young adults do. In the right panel of Figure 1, we can observe this in that the categorization proportions of the older adults tend to be higher than the categorization proportions of the young adults. Some proportion differences are more or less pronounced than one would expect based on this degree difference. The corresponding items were identified as differently functioning items in the model analysis (indicated by arrows). The categorization difference for the items 14 and 17 (corresponding to the items *sea horse* and *squid*) was more pronounced than one would expect based on the degree difference alone. Items 10 and 11 (corresponding to *jellyfish* and *plankton*) were more endorsed by young adults and thus defy the degree difference.

## 5. General Discussion

### 5.1. Main findings

Whether there are age differences in semantic structure has been studied far less often than whether or not healthy aging affects semantic processing, such as in semantic priming or picture-word interference tasks (e.g., Laver, 2009; Laver & Burke, 1993; Spieler & Balota, 2000; Taylor & Burke, 2002). This is somewhat surprising since the study of age differences in semantic structure in a sense precedes the study of age differences in semantic processing. One would like to ensure that young and older adults rely on the same semantic information to ensure that any group differences can be unequivocally interpreted as process differences (e.g., Balota, Cortese, Sergent-Marshall, Spieler, & Yap, 2004; Balota & Duchek, 1988; De Deyne & Storms, 2007; Dorot & Mathey, 2010; Fitzpatrick, Playfoot, Wray, & Wright, 2013; Morrow & Duffy, 2005; Rönnlund, Nyberg, Bäckman, & Nilsson, 2005).

The current study shows the importance of matching the materials used in studies that compare the semantic processing of young and older adults. In a semantic categorization task, young and older adults were shown to differ both with respect to the conditions for application of common language terms (vagueness in criteria) and with respect to the extent to which these terms can be applied given fixed conditions (vagueness in degree). Older adults tended to have larger category extensions than young adults and the endorsement probability of individual items could differ markedly between groups. Since these findings are based on binary categorization data rather than on ratings, they cannot be due to differences in scale use. They are not a consequence of cognitive slowing either as the categorization task was untimed and participants could take as much time as they wanted to respond. Nor are the differences due to gender or education differences, because these factors were controlled for in our analyses. The established differences need to be taken into account when interpreting semantic processing differences between age groups, since differences in the membership status of items are likely to affect the items’ behavior in a range of semantic tasks (e.g., through the extent to which they might or might not be perceived as related to other instances in priming or picture-word interference paradigms).

A formal model that not only takes into account individual categorization differences, but is also able to distinguish the nature of group differences in categorization, was key to obtaining the above results. The RIM model (Frederickx, Tuerlinckx, De Boeck, & Magis, 2010) can be considered a formalization of the Threshold Theory (Hampton, 1998, 2007) which accounts for semantic categorization differences in terms of distinct assessments of items with respect to categorization criteria and the use of distinct thresholds on said criteria to establish category membership. The analyses with the RIM model allowed us to establish when items were being categorized differently because of a different assessment of the categorization criteria and/or the use of different thresholds by older and young adults. This could not be achieved by merely comparing categorization proportions, since they entangle criteria and degree differences (Stukken, Verheyen, & Storms, 2013; Verheyen & Storms, 2018).

We undertook this study after coming across a paper by Morrow and Duffy on semantic memory in healthy old age. Morrow and Duffy (2005) reported that older adults on average provided higher typicality ratings than young adults did. Despite this difference, the typicality ratings elicited from both groups correlated highly, albeit imperfectly. The current study suggests that the mean rating difference could reflect the use of a lower membership threshold by the older participants compared to the younger group. Across a range of semantic categories, we found older adults to be more lenient categorizers than young adults. Like Morrow and Duffy, we observed that older and young adults tended to agree on the items’ representativeness for the target categories. The posterior means of the item positions *β_i_* along the latent dimensions used by older and young adults correlated strongly. This can also be observed in Figure 1, which depicts the proportions of category endorsement in the two groups (see section 4.3 Categorization patterns): The categorization proportions of older adults follow more or less the same trend as the categorization proportions of the young adults. The imperfect typicality correlations can be explained by the observation of differentially functioning items in the young and the older groups: some items are considered more representative of the target category in the older group than in the young group (and vice versa). Note that we do not expect these differences to be very pronounced. The use of completely distinct criteria by different groups in the same language community would hamper their communication (Hampton & Passanisi, 2016; Verheyen & Storms, 2013). The consistency with which many items are used and the restricted range of contexts in which they are encountered might also make them less prone to differences (Verheyen, Heussen, & Storms, 2011).

Our findings go against the prevailing view in the older literature that the semantic structure of adults of different ages does not differ (e.g., Burke & Peters, 1986; Mayr & Kliegl, 2000), but does not contradict the idea that semantic structure does not change with age (e.g., Light, 1991). The structure of common categories could become entrenched early in life and remain fairly immune to change over time (Thiessen, Girard, & Erickson, 2016). The differences we observed could then be due to differences in the early life experiences of the different cohorts (Yoon et al. 2004; see also Howard, 1980). This explanation is, for instance, put forward to account for the observation in White et al. (2018) that older adults who grew up with glass bottles continue to consider glass bottles more representative of the category BOTTLES than plastic ones, despite the fact that plastic bottles are presently more in use and younger adults consider them prototypical examples of BOTTLES.

### 5.2. Explanation of differences

While the current study establishes that there are meaningful degree and criteria differences between younger and older adults, it does not offer an explanation of why these differences arise. The study was first and foremost intended to investigate whether age-related degree and criteria differences in semantic categorization exist. We therefore opted to use materials that were used in previous studies on individual and group differences in language term use. Our results can nevertheless be used to inform hypotheses about the reasons for the established differences, which can then be tested in new studies in which the stimuli are selected with respect to the proposed explanations.

#### 5.2.1. Degree differences

We hypothesized that older adults would use a lower threshold for semantic categorization than young adults *across* categories. While we only established such a reliable degree difference in 2 out of the 8 studied categories, the effect was in the proposed direction in the majority of categories and participant groups we studied (contrary to what one would expect if there were no systematic age differences; see Appendix B for a simulation study). The observation that older adults on average tend to entertain broader category extensions than younger adults do, is on par with the conception of the older semantic network by Buchler and Reder (2007) in which concepts are more easily activated due to the combination of a higher resting level of activation and a more diffuse spread of activation. These network characteristics, in turn, are said to be the result of older adults acquiring more stable and more extended word representations throughout their longer lives relative to younger adults (Morrow & Duffy, 2005; see also Gilet et al. 2012). Without external validation, we are reluctant to explain the varying magnitude of the degree difference between our categories in terms of varying differences in experience with the respective domains. Instead, we propose that any future studies also include categories older adults are presumably less experienced with than younger adults and to see whether the direction of the degree difference reverses.

The observation that there are categories for which older adults use a higher threshold for semantic categorization than young adults would also allow one to exclude the possibility that both the observed degree differences in our study and the mean typicality rating difference in Morrow and Duffy (2005) are due to an increased acquiescence bias with age. Both in brand attitude measurements (Jayanti, McManamon, & Whipple, 2004) and survey studies (Lechner, Partsch, Danner, & Rammstedt, 2019; Meisenberg & Williams, 2008; Weijters, Geuens, & Schillewaert, 2010) older adults have been found to display more favorable attitudes and to agree more with statements, regardless of their contents (but see Eid & Rauber, 2000, and Rammstedt, Danner, & Bosnjak, 2017, for contradictory evidence). An explanation in terms of increased acquiescence bias is supported by the observation that Morrow and Duffy found comparable rating differences between young and older adults for variables that do not appear to pertain to the categories’ extension, such as visual complexity and imageability. The latter differences are less straightforwardly accounted for in terms of increased experience in older adults than differences in typicality or semantic categorization are. We do not know of a study that has explicitly investigated whether the scales for lexico-semantic variables are suspect to increased acquiescence bias with age, but researchers interested in composing age-specific norms (see below) are advised to keep potential response biases due to interactions of age with scale in mind when setting up their studies (see Jayanti, McManamon, & Whipple, 2004, for recommendations).

#### 5.2.2. Criteria differences

Duration is not the only factor affecting one’s experience with categories. Because they have different interests and engage in different activities, the salience of some categories may also differ between older and younger adults (Yoon et al. 2004). We did not observe categories for which young and older adults relied on completely distinct criteria, however. Rather, the criteria differences we observed pertained to individual instances, suggesting that inequalities in salience tend to present at the item level. Individuals from different age groups would then assess the standing of instances along the categorization criterion differently due to cohort differences in familiarity and/or age of acquisition, affecting the knowledge and/or beliefs they have about these instances (De Deyne & Storms, 2007; Little, Prentice, & Wingfield, 2004; Morrow & Duffy, 2005; Pennequin, Fontaine, Bonthoux, Scheuner, & Blaye, 2006).

One’s familiarity with an item is known to influence how representative it is considered of its category (Janczura & Nelson, 1999; Johnson, 2001). Items are judged to be more representative category members, the more frequently one encounters them and the more acquainted one is with them. Familiarity could thus make items relatively more endorsed by older or by younger adults, depending on whether they are more familiar to older or to younger adults. Some of the items that functioned differently in the two age groups in our study seem to suggest that this is a feasible interpretation. The older male participants, for instance, found *hunting* to be more representative of SPORTS than the young participants did. In the female sample, the older participants found *aerobics* to be a less representative example of SPORTS than the young participants did.

The age at which some words are first learned can differ considerably between older and young adults (De Deyne & Storms, 2007; Morrow & Duffy, 2005). Morrow and Duffy (2005, p. 615) attribute these cohort differences in age of acquisition to (i) technological innovations (e.g., for TOOLS), (ii) the availability of foreign imports (e.g., for FRUIT), (iii) increased travel opportunities, and (iv) the increased availability of information through various types of media (e.g., allowing people to learn about nonnative FISH). These age of acquisition differences, in turn, can affect semantic categorization. The effect of a later age of acquisition due to technological innovation was already mentioned in the introduction: Malt and Paquet (2013) found that younger adults judged recently introduced objects such as a *cell phone* or an *electronic swipe* to really be PHONES or KEYS, while older adults disagreed. Some of the items that functioned differently in the two age groups in our study appear to support the effect of changes in the range of FRUIT available to participants. The older male adults regarded *pomegranates* less typical of FRUIT than the younger adults did, for instance, while the reverse held for *walnuts*.

One might inspect the instances for which criteria differences did and did not arise in our study (see Appendix A) to test whether these explanations apply and/or to come up with additional explanations. Since these investigations would require the collection of extensive age-specific norm data, we defer them to future research. Our findings, combined with the explanations offered for the various differences, advocate the use of age-specific norms for lexico-semantic research (see, for instance, De Deyne & Storms, 2007; Gilet, Grühn, Studer, & Labouvie-Vief, 2012; Gobin, Camblats, Faurous, & Mathey, 2017; Göz, Tekcan, & Erciyes, 2017; Grühn & Smith, 2008; Morrow & Duffy, 2005; Söderholm, Häyry, Laine, & Karrasch, 2013).

### 5.3. Education differences

We found that degree differences in categorization were more pronounced when we did not control for education level. The data from all of the female participants showed more categories with a reliable degree difference than the female data that were equated for education level. Since the older participants were found to maintain lower thresholds for categorization than the younger ones, and the young female adults in our sample were more highly educated compared to the older female adults (see section 3.1 Participants), this finding suggests that more highly educated individuals entertain higher thresholds for semantic categorization. One interpretation, suggested in Verheyen and Storms (2018), is that highly educated individuals reject more semantic foils because their deliberations are more thorough and deliberate because they tend to be more conscientious (Denissen, Geenen, van Aken, Gosling, & Potter, 2008). This interpretation is also consistent with an increased acquiescence among lower educated individuals (e.g., Lechner, Partsch, Danner, & Rammstedt, 2019; Meisenberg & Williams, 2008; Rammstedt, Danner, & Bosnjak, 2017; Weijters, Geuens, & Schillewaert, 2010).

We did not find an effect of education level on criteria differences. The number of differently functioning items did not systematically differ between the data sets that were and were not equated on education level. This result is line with that of Verheyen and Storms (2018) in which hardly any criteria differences were found between the semantic categorization data of participants who went on to higher education after completing compulsory education and participants who did not. It also seems to speak against the hypothesis that the criteria differences that were found between young and older adults are due to the young, higher educated individuals relying more on rules (such as the biological rule <has six legs> for the natural category INSECTS) than the older, lower educated individuals. Verheyen and Storms (2018) found that highly educated individuals employ entirely different application conditions for terms they are more familiar with through schooling (i.e., SCIENCES), while we find that the criteria differences take the form of a different assessment of individual instances with respect to comparable conditions. That is not to say that the different assessment of individual instances cannot be due to knowledge differences that stem from a different educational background (see also section 5.2.2).

We propose that future studies undertake a more systematic investigation of the interaction between age and education differences. Such studies could also investigate to what extent our findings generalize to lower educated participants, seeing that the participants in our study were predominantly highly educated (64% continued education after secondary education).

## 6. Conclusion

We analyzed the semantic categorization data of nearly 2,000 young and older adults with a statistical model that allows group differences to be qualified as degree and/or criteria differences. Our results indicate that older adults maintain somewhat lower thresholds for category membership than young adults do (degree difference). We identified individual items with a considerable higher or lower probability of being considered category members by the older than by the young adults, indicating that these items were differently assessed with respect to the categorization criteria used by older and young adults (criteria differences). These findings indicate that studies on age-related semantic processing should recognize the age-specific nature of semantic representations.

## Data Accessibility Statement

The data and exemplary model code can be found on the Open Science Framework (DOI 10.17605/OSF.IO/TBVZ8). The data can be consulted at https://osf.io/yt7mf/. Exemplary code can be found at https://osf.io/8ejbw/. The data have been previously reported on in Verheyen, S., & Storms, G. (2018). Education as a source of vagueness in criteria and degree. In E. Castroviejo, L. McNally, & G. W. Sassoon (Eds.), *The Semantics of Gradability, Vagueness, and Scale Structure: Experimental Perspectives* (pp. 149–167). Berlin, Germany: Springer.

## Additional Files

The additional files for this article can be found as follows:

10.5334/joc.74.s1 Appendix A. Overview of the materials in English (materials were presented in Dutch). Indices *m, f*, and *e* indicate items that function differently in young and older adults for the Male, Female, and Female Education Equated data, respectively. Superscripted indices indicate items that were more often endorsed by the older than by the young adults. Subscripted indices indicate items that were more often endorsed by the young than by the older adults.

10.5334/joc.74.s2 Appendix B. Monte Carlo Simulations.

## Footnotes

We acknowledge that the term criterial vagueness appears to apply better to the use of different substantive criteria than to the different assessment of individual exemplars with respect to categorization criteria, and that for some purposes it could be interesting to discern these cases, perhaps even in name. Stukken, Verheyen, and Storms (2013) coined the term ‘representational vagueness’ for the latter case. Here we choose, however, to use ‘criterial vagueness’ for both cases, since formally they present in the same manner – as differences in item positions between groups – and discerning them would require additional information (e.g., evidence that the differently functioning items share some common feature which is being given variable weight, to identify the differences as criterial vagueness as opposed to representational vagueness).

The decision which age ranges constitute the young and older group is somewhat arbitrary. We ran additional analyses with groups with a more pronounced age difference. This tended to make the degree and criteria differences between the age groups more pronounced, but also less reliable since they are necessarily based on a smaller number of participants.

We do not regard the semantic categorization task superior to other tasks that tap into semantic representations. No task is process pure and all tasks have their merits and costs. The semantic categorization task does not allow one to make the subtle distinctions between category exemplars the typicality rating task affords, for instance. It would identify both *sparrows* and *penguins* as members of the BIRDS category, but would not signal that *sparrows* are more typical BIRDS than *penguins* are. We do believe the semantic categorization task is particularly suited to establish the extension of categories.

We also compared threshold variability in young vs. older adults by determining the 95% credibility interval for {\sigma _{{\theta _Y}}} - {\sigma _{{\theta _O}}}, the difference of the group-specific standard deviations of the thresholds distributions. Only in 3 out of 3 × 8 comparisons did the 95% credibility interval not include zero (more variable thresholds in young than in older adults for TOOLS in the Female data and for VEGETABLES in the Male data; more variable thresholds in older than in young adults for FRUITS in the Male data). The median difference was negative in 3 out of 8 categories in the Female and Female Education Equated data and in 4 out of 8 categories in the Male data. It thus appears that there is no systematic relationship between age and threshold variability.

The observation that the number of items endorsed more by the older group is more or less matched by the number of items endorsed more by the younger group is not a necessary consequence of the threshold matching that is part of the identification of differently functioning items (see Frederickx et al. 2010, for counterevidence and discussion), but presumably the result of the random sampling of stimulus materials, which is likely to include both types of items.

## References

[1] Alston, W. P. (1964). Philosophy of Language. Englewood Cliffs: Prentice-Hall.

[2] Annett, M. (1959). The classification of instances of four common class concepts by children and adults. British Journal of Educational Psychology, 29, 223–236. DOI: 10.1111/j.2044-8279.1959.tb01503.x

[3] Balota, D. A., Cortese, M. J., Sergent-Marshall, S. D., Spieler, D. H., & Yap, M. J. (2004). Visual word recognition of single-syllable words. Journal of Experimental Psychology: General, 133, 283–316. DOI: 10.1037/0096-3445.133.2.283 15149254

[4] Balota, D. A., & Duchek, J. M. (1988). Age-related differences in lexical access, spreading activation, and simple pronunciation. Psychology and Aging, 3, 84–93. DOI: 10.1037//0882-7974.3.1.84 3268246

[5] Black, M. (1937). Vagueness: An exercise in logical analysis. Philosophy of Science, 4, 427–455. DOI: 10.1086/286476

[6] Borel, E. (1907). Un paradoxe économique: Le sophisme du tas de blé et les vérités statistiques. La Revue du Mois, 4, 688–699. [English translation by Égré, P., & Gray, E. (2014). An economic paradox: The sophism of the heap of wheat and statistical truths. *Erkenntnis, 79*, 1081–1088. DOI: 10.1007/s10670-014-9615-z]

[7] Brooks, S. P., & Gelman, A. (1998). General methods for monitoring convergence of iterative simulations. Journal of Computational and Graphical Statistics, 7, 434–455. DOI: 10.1080/10618600.1998.10474787

[8] Brosseau, J., & Cohen, H. (1996). The representation of semantic categories in aging. Experimental Aging Research, 22, 381–391. DOI: 10.1080/03610739608254018 8968709

[9] Buchler, N. E. G., & Reder, L. M. (2007). Modeling age-related memory deficits: A two-parameter solution. Psychology and Aging, 22, 104–121. DOI: 10.1037/0882-7974.22.1.104 17385988

[10] Burke, D., & Peters, L. (1986). Word association in old age: Evidence for consistency in semantic encoding during adulthood. Psychology and Aging, 1, 283–292. DOI: 10.1037//0882-7974.1.4.283 3267408

[11] Burks, A. W. (1946). Empiricism and vagueness. Journal of Philosophy, 43, 477–486. DOI: 10.2307/2018954

[12] Carlson, M. C., Hasher, L., Connelly, S. L., & Zacks, R. T. (1995). Aging, distraction, and the benefits of predictable location. Psychology and Aging, 10, 427–436. DOI: 10.1037//0882-7974.10.3.427 8527063

[13] Cicirelli, V. G. (1976). Categorization behavior in aging subjects. Journal of Gerontology, 31, 676–680. DOI: 10.1093/geronj/31.6.676 977926

[14] Collins, A. M., & Loftus, E. F. (1975). A spreading-activation theory of semantic processing. Psychological Review, 82, 407–428. DOI: 10.1037//0033-295x.82.6.407

[15] Collins, A. M., & Quillian, M. R. (1969). Retrieval time from semantic memory. Journal of Verbal Learning and Verbal Behavior, 8, 240–247. DOI: 10.1016/s0022-5371(69)80069-1

[16] Conley, P., & Burgess, C. (2000a). Age effects in a computational model of memory. Brain & Cognition, 43, 104–108. DOI: 10.1016/s0278-2626(99)91133-8 10857673

[17] Conley, P., & Burgess, C. (2000b). A computational approach to modeling population differences. Behavior Research Methods, Instruments, & Computers, 32, 274–279. DOI: 10.3758/bf03207795 10875174

[18] De Deyne, S., & Storms, G. (2007). Age-of-acquisition differences in young and older adults affect latencies in lexical decision and semantic categorization. Acta Psychologica, 124, 274–295. DOI: 10.1016/j.actpsy.2006.03.007 16777041

[19] Denissen, J. J. A., Geenen, R., van Aken, M. A. G., Gosling, S. D., & Potter, J. (2008). Development and validation of a Dutch translation of the Big Five Inventory (BFI). Journal of Personality Assessment, 90, 152–157. DOI: 10.1080/00223890701845229 18444109

[20] Devos, F. (1995). Still fuzzy after all these years. A linguistic evaluation of the fuzzy set approach to semantic vagueness. Quaderni di Semantica, 16, 47–82.

[21] Devos, F. (2003). Semantic vagueness and lexical polyvalence. Studia Linguistica, 57, 121–141. DOI: 10.1111/j.0039-3193.2003.00101.x

[22] Dorot, D., & Mathey, S. (2010). Visual word recognition in young and older adults: A study of cohort effects for lexical variables. Revue Européenne de Psychologie Appliquée/European Review of Applied Psychology, 60, 163–172. DOI: 10.1016/j.erap.2010.02.001

[23] Dubossarsky, H., De Deyne, S., & Hills, T. (2017). Quantifying the structure of free association networks across the lifespan. Developmental Psychology, 53, 1560–1570. DOI: 10.1037/dev0000347 28569517

[24] Eid, M., & Rauber, M. (2000). Detecting measurement invariance in organizational surveys. European Journal of Psychological Assessment, 16, 20–30. DOI: 10.1027//1015-5759.16.1.20

[25] Embretson, S. E., & Reise, S. P. (2000). Item Response Theory for Psychologists. Mahwah, NJ: Lawrence Erlbaum DOI: 10.4324/9781410605269

[26] Fitzpatrick, T., Playfoot, D., Wray, A., & Wright, M. J. (2013). Establishing the reliability of word association data for investigating individual and group differences. Applied Linguistics, 36, 1–29. DOI: 10.1093/applin/amt020

[27] Frederickx, S., Tuerlinckx, F., De Boeck, P., & Magis, D. (2010). RIM: A random item mixture model to detect differential item functioning. Journal of Educational Measurement, 47, 432–457. DOI: 10.1111/j.1745-3984.2010.00122.x

[28] Gelman, A., & Hill, J. (2007). Data analysis using regression and multilevel/hierarchical models. Cambridge, UK: Cambridge University Press DOI: 10.1017/CBO9780511790942

[29] Giffard, B., Desgranges, B., Kerrouche, N., Piolino, P., & Eustache, F. (2003). The hyperpriming phenomenon in normal aging: A consequence of cognitive slowing? Neuropsychology, 17, 594–601. DOI: 10.1037/0894-4105.17.4.594 14599272

[30] Gilet, A.-L., Grühn, D., Studer, J., & Labouvie-Vief, G. (2012). Valence, arousal, and imagery ratings for 835 French attributes by young, middle-aged, and older adults: The French Emotional Evaluation List (FEEL). Revue Européenne de Psychologie Appliquée/European Review of Applied Psychology, 62, 173–181. DOI: 10.1016/j.erap.2012.03.003

[31] Gobin, P., Camblats, A.-M., Faurous, W., & Mathey, S. (2017). A base of emotionality (valence, arousal, category) of 1286 French words according to age (EMA). Revue Européenne de Psychologie Appliquée/European Review of Applied Psychology, 67, 25–42. DOI: 10.1016/j.erap.2016.12.001

[32] Göz, I., Tekcan, A. I., & Erciyes, A. A. (2017). Subjective age-of-acquisition norms for 600 Turkish words from four age groups. Behavior Research Methods, 49, 1736–1746. DOI: 10.3758/s13428-016-0817-y 27743317

[33] Grühn, D., & Smith, J. (2008). Characteristics for 200 words rated by young and older adults: Age-dependent evaluations of German adjectives (AGE). Behavior Research Methods, 40, 1088–1097. DOI: 10.3758/brm.40.4.1088 19001400

[34] Hampton, J. A. (1995). Testing the prototype theory of concepts. Journal of Memory and Language, 34, 686–708. DOI: 10.1006/jmla.1995.1031

[35] Hampton, J. A. (1998). Similarity-based categorization and fuzziness of natural categories. Cognition, 65, 137–165. DOI: 10.1016/s0010-0277(97)00042-5 9557381

[36] Hampton, J. A. (2006). Concepts as prototypes. The Psychology of Learning and Motivation: Advances in Research and Theory, 46, 79–113. DOI: 10.1016/s0079-7421(06)46003-5

[37] Hampton, J. A. (2007). Typicality, graded membership, and vagueness. Cognitive Science, 31, 355–384. DOI: 10.1080/15326900701326402 21635301

[38] Hampton, J. A., Dubois, D., & Yeh, W. (2006). Effects of classification context on categorization in natural categories. Memory & Cognition, 34, 1431–1443. DOI: 10.3758/bf03195908 17263068

[39] Hampton, J. A., & Gardiner, M. M. (1983). Measures of internal category structure: A correlational analysis of normative data. British Journal of Psychology, 74, 491–516. DOI: 10.1111/j.2044-8295.1983.tb01882.x

[40] Hampton, J. A., & Passanisi, A. (2016). When intensions do not map onto extensions: Individual differences in conceptualization. Journal of Experimental Psychology: Learning, Memory, & Cognition, 42, 505–523. DOI: 10.1037/xlm0000198 26551627

[41] Hasher, L., Zacks, R. T., & May, C. P. (1999). Inhibitory control, circadian arousal and age In D. Gopher, & A. Koriat (Eds.), Attention & performance XVII: Cognitive regulation and performance: Interaction of theory and application (pp. 653–675). Cambridge, MA: MIT Press.

[42] Hirsh, K. W., & Tree, J. J. (2001). Word association norms for two cohorts of British adults. Journal of Neurolinguistics, 14, 1–44. DOI: 10.1016/s0911-6044(00)00002-6

[43] Howard, D. V. (1980). Category norms: A comparison of the Battig and Montague (1969) norms with the responses of adults between the ages of 20 and 80. Journal of Gerontology, 35, 225–231. DOI: 10.1093/geronj/35.2.225 7410780

[44] Howard, D. V. (1983). A multidimensional scaling analysis of aging and the semantic structure of animal names. Experimental Aging Research, 9, 27–30. DOI: 10.1080/03610738308258416 6861836

[45] Janczura, G. A., & Nelson, D. L. (1999). Concept accessibility as the determinant of typicality judgments. The American Journal of Psychology, 112, 1–19. DOI: 10.2307/1423622

[46] Jayanti, R. K., McManamon, M. K., & Whipple, T. W. (2004). The effects of aging on brand attitude measurement. Journal of Consumer Marketing, 21, 264–273. DOI: 10.1108/07363760410542174

[47] Johnson, K. E. (2001). Impact of varying levels of expertise on decisions of category typicality. Memory & Cognition, 29, 1036–1050. DOI: 10.3758/bf03195765 11820747

[48] Kempton, W. (1981). The folk classification of ceramics: A study of cognitive prototypes. New York: Academic Press DOI: 10.1016/b978-0-12-404080-9.x5001-7

[49] Kennedy, C. (2013). Two sources of subjectivity: Qualitative assessment and dimensional uncertainty. Inquiry, 56, 258–277. DOI: 10.1080/0020174x.2013.784483

[50] Kogan, N. (1974). Categorizing and conceptualizing styles in younger and older adults. Human Development, 17, 218–230. DOI: 10.1159/000271345 4412352

[51] Kölbel, M. (2004). Faultless disagreement. Proceedings of the Aristotelian Society, 104, 53–73. DOI: 10.1111/j.0309-7013.2004.00081.x

[52] Laver, G. D. (2009). Adult aging effects on semantic and episodic priming in word recognition. Psychology & Aging, 24, 28–39. DOI: 10.1037/a0014642 19290735

[53] Laver, G. D. & Burke, D. M. (1993). Why do semantic priming effects increase in old age? A meta-analysis. Psychology and Aging, 8, 34–43. DOI: 10.1037//0882-7974.8.1.34 8461113

[54] Lechner, C. M., Partsch, M. V., Danner, D., & Rammstedt, B. (2019). Individual, situational, and cultural correlates of acquiescent responding: Towards a unified conceptual framework. British Journal of Mathematical and Statistical Psychology. DOI: 10.1111/bmsp.12164 30851072

[55] Light, L. L. (1991). Memory and aging: Four hypotheses in search of data. Annual Review of Psychology, 42, 333–376. DOI: 10.1146/annurev.ps.42.020191.002001 2018397

[56] Light, L. L. (2000). Memory changes in adulthood In S. H. Qualls, & N. Abeles (Eds.), Psychology and the aging revolution: How we adapt to longer life (pp. 73–97). Washington, DC: American Psychological Association DOI: 10.1037/10363-005

[57] Little, D. M., Prentice, K. J., & Wingfield, A. (2004). Adult age differences in judgments of semantic fit. Applied Psycholinguistics, 25, 135–143. DOI: 10.1017/s0142716404001079

[58] Lovelace, E. A., & Cooley, S. (1982). Free associations of older adults to single words and conceptually related word triads. Journal of Gerontology, 37, 432–437. DOI: 10.1093/geronj/37.4.432 7086079

[59] Lunn, D. J., Thomas, A., Best, N., & Spiegelhalter, D. (2000). WinBUGS: A Bayesian modeling framework: Concepts, structure, and extensibility. Statistics and Computing, 10, 325–337. DOI: 10.1023/a:1008929526011

[60] Machina, K. F. (1976). Truth, belief and vagueness. Journal of Philosophical Logic, 5, 47–78. DOI: 10.1007/bf00263657

[61] Malt, B. C., & Paquet, M. R. (2013). The real deal: What judgments of really reveal about how people think about artifacts. Memory and Cognition, 41, 354–364. DOI: 10.3758/s13421-012-0270-9 23138566

[62] Mayr, U., & Kliegl, R. (2000). Complex semantic processing in old age: does it stay or does it go? Psychology and Aging, 15, 29–43. DOI: 10.1037//0882-7974.15.1.29 10755287

[63] McCloskey, M. E., & Glucksberg, S. (1978). Natural categories: Well defined or fuzzy sets? Memory & Cognition, 6, 462–472. DOI: 10.3758/bf03197480

[64] Meisenberg, G., & Williams, A. (2008). Are acquiescent and extreme response styles related to low intelligence and education? Personality and Individual Differences, 44, 1539–1550. DOI: 10.1016/j.paid.2008.01.010

[65] Mervis, C. B., Catlin, J., & Rosch, E. (1976). Relationships among goodness-of-example, category norms, and word frequency. Bulletin of the Psychonomic Society, 7, 283–284. DOI: 10.3758/bf03337190

[66] Morrow, L. A., & Duffy, M. F. (2005). The representation of ontological category concepts as affected by healthy aging: Normative data and theoretical implications. Behavior Research Methods, 37, 608–625. DOI: 10.3758/bf03192731 16629293

[67] Myerson, J., Hale, S., Chen, J., & Lawrence, B. (1997). General lexical slowing and the semantic priming effect: The roles of age and ability. Acta Psychologica, 96, 83–101. DOI: 10.1016/s0001-6918(97)00002-4 9210852

[68] Pennequin, V., Fontaine, R., Bonthoux, F., Scheuner, N., & Blaye, A. (2006). Categorization deficit in old age: Reality or artefact? Journal of Adult Development, 13, 1–9. DOI: 10.1007/s10804-006-9000-5

[69] Phillips, L. H. (1999). Age and individual differences in letter fluency. Developmental Neuropsychology, 15, 249–267. DOI: 10.1080/87565649909540748

[70] Raffman, D. (2014). Unruly words: A study of vague language. New York, NY: Oxford University Press DOI: 10.1093/acprof:oso/9780199915101.001.0001

[71] Rammstedt, B., Danner, D., & Bosnjak, M. (2017). Acquiescence response styles: A multilevel model explaining individual-level and country-level differences. Personality and Individual Differences, 107, 190–194. DOI: 10.1016/j.paid.2016.11.038

[72] Rönnlund, M., Nyberg, L., Bäckman, L., & Nilsson, L.-G. (2005). Stability, growth, and decline in adult life span development of declarative memory: Cross-sectional and longitudinal data from a population-based study. Psychology and Aging, 20, 3–18. DOI: 10.1037/0882-7974.20.1.3 15769210

[73] Scialfa, C. T., & Margolis, R. B. (1986). Age differences in the commonality of free associations. Experimental Aging Research, 12, 95–98. DOI: 10.1080/03610738608259443 3569391

[74] Smiley, S. S., & Brown, A. L. (1979). Conceptual preference for thematic or taxonomic relations: A nonmonotonic age trend from preschool to old age. Journal of Experimental Child Psychology, 28, 249–257. DOI: 10.1016/0022-0965(79)90087-0

[75] Söderholm, C., Häyry, E., Laine, M., & Karrasch, M. (2013). Valence and arousal ratings for 420 Finnish nouns by age and gender. PLoS ONE, 8(8), e72859 DOI: 10.1371/journal.pone.0072859 24023650 PMC3758333

[76] Spieler, D. H., & Balota, D. A. (2000). Factors influencing word naming in younger and older adults. Psychology and Aging, 15, 225–231. DOI: 10.1037//0882-7974.15.2.225 10879577

[77] Stukken, L., Verheyen, S., & Storms, G. (2013). Representation and criterion differences between men and women in semantic categorization. In M. Knauff, M. Pauen, N. Sebanz, & I. Wachsmuth (Eds.), Proceedings of the 35th Annual Conference of the Cognitive Science Society (pp. 3474–3479). Austin, TX: Cognitive Science Society.

[78] Taylor, J. K., & Burke, D. M. (2002). Asymmetric aging effects on semantic and phonological processes: Naming in the picture-word interference task. Psychology and Aging, 17, 662–676. DOI: 10.1037//0882-7974.17.4.662 12507362

[79] Thiessen, E. D., Girard, S., & Erickson, L. C. (2016). Statistical learning and the critical period: How a continuous learning mechanism can give rise to discontinuous learning. Cognitive Science, 7, 276–288. DOI: 10.1002/wcs.1394 27239798

[80] Tresselt, M. E., & Mayzner, M. S. (1964). The Kent-Rosanoff word association: Word association norms as a function of age. Psychonomic Science, 1, 65–66. DOI: 10.3758/bf03342792

[81] Verheyen, S., Ameel, E., & Storms, G. (2011). Overextensions that extend into adolescence: Insights from a threshold model of categorization. In L. Carlson, C. Hölscher, & T. F. Shipley (Eds.), Proceedings of the 33rd Annual Conference of the Cognitive Science Society (pp. 2000–2005). Austin, TX: Cognitive Science Society.

[82] Verheyen, S., De Deyne, S., Dry, M. J., & Storms, G. (2011). Uncovering contrast categories in categorization with a probabilistic threshold model. Journal of Experimental Psychology: Learning, Memory, and Cognition, 37, 1515–1531. DOI: 10.1037/a0024431 21767056

[83] Verheyen, S., Dewil, S., & Egré, P. (2018). Subjectivity in gradable adjectives: The case of *tall* and *heavy*. Mind & Language, 1–20. DOI: 10.1111/mila.12184

[84] Verheyen, S., Hampton, J. A., & Storms, G. (2010). A probabilistic threshold model: Analyzing semantic categorization data with the Rasch model. Acta Psychologica, 135, 216–225. DOI: 10.1016/j.actpsy.2010.07.002 20682453

[85] Verheyen, S., Heussen, D., & Storms, G. (2011). On domain differences in categorization and context variety. Memory & Cognition, 39, 1290–1300. DOI: 10.3758/s13421-011-0102-3 21538180

[86] Verheyen, S., & Storms, G. (2013). A mixture approach to vagueness and ambiguity. PLoS ONE, 8(5): e63507 DOI: 10.1371/journal.pone.0063507 23667627 PMC3646747

[87] Verheyen, S., & Storms, G. (2018). Education as a source of vagueness in criteria and degree In E. Castroviejo, L. McNally, & G. W. Sassoon (Eds.), The Semantics of Gradability, Vagueness, and Scale Structure: Experimental Perspectives (pp. 149–167). Berlin, Germany: Springer DOI: 10.1007/978-3-319-77791-7_6

[88] Verheyen, S., Voorspoels, W., & Storms, G. (2015). Inferring choice criteria with mixture IRT models: A demonstration using ad hoc and goal-derived categories. Judgment and Decision Making, 10, 97–114.

[89] Weijters, B., Geuens, M., & Schillewaert, N. (2010). The stability of individual response styles. Psychological Methods, 15, 96–110. DOI: 10.1037/a0018721 20230106

[90] White, A., Storms, G., Malt, B. C., & Verheyen, S. (2018). Mind the generation gap: Differences between young and old in the representation of everyday objects. Journal of Memory and Language, 98, 12–25. DOI: 10.1016/j.jml.2017.09.001

[91] Wright, C. (1995). The epistemic conception of vagueness. The Southern Journal of Philosophy, 33, 133–159. DOI: 10.1111/j.2041-6962.1995.tb00767.x

[92] Yoon, C., Feinberg, F., Hu, P., Gutchess, A. H., Hedden, T., Chen, H.-Y. M., Jing, Q., & Cui, Y. (2004). Category norms as a function of culture and age: Comparisons of item responses to 105 categories by American and Chinese adults. Psychology and Aging, 19, 379–393. DOI: 10.1037/0882-7974.19.3.379 15382989

[93] Zortea, M., Menegola, B., Villavicencio, A., & Salles, J. F. (2014). Graph analysis of semantic word association among children, adults, and the elderly. Psicologia: Reflexão e Crítica, 27, 90–99. DOI: 10.1590/s0102-79722014000100011

