# Supplementary material for: Age-Related Degree and Criteria Differences in Semantic Categorization
Source: J Cogn. 2019 Jul 19;2(1):17. doi: 10.5334/joc.74 (PMC6640261; doi:10.5334/joc.74)
Supplement: Appendix A. — Overview of the materials in English (materials were presented in Dutch). Indices m, f, and e indicate items that function differently in young and older adults for the Male, Female, and Female Education Equated data, respectively. Superscripted indices indicate items that were more often endorsed by the older than by the young adults. Subscripted indices indicate items that were more often endorsed by the young than by the older adults. [file joc-2-1-74-s1.pdf]

Appendix A: Overview of the materials in English (materials were presented in Dutch). Indices *m*, *f*, and *e* indicate items that function differently in young and older adults for the Male, Female, and Female Education Equated data, respectively. Superscripted indices indicate items that were more often endorsed by the older than by the young adults. Subscripted indices indicate items that were more often endorsed by the young than by the older adults.

FISH	INSECTS	FURNITURE	TOOLS	FRUIT	VEGETABLES	SCIENCES	SPORTS
alligator	amoeba	ashtray	axe	acorn	apple	advertising	aerobics$_{f,e}$
catfish	ant	bed	broom^{f}	almond^{m,e}	artichoke	agriculture	ballroom dancing$_{f,e}$
clam	bacterium	book	calculator	avocado	asparagus	archaeology	billiards
crab	bat	bookends	dictionary	banana	bamboo shoot	architecture	bridge
eel	caterpillar	bucket	funnel	carrot	bread	astrology^{m}	bullfighting
frog	centipede	chair	hammer	coconut$_{f}$	celery	astronomy	chess
goldfish	dust mite^{m,f,e}	curtains	key	cucumber	cereal	chemistry	conversation
gull	earthworm$_{m,f,e}$	cushion$_{m,f,e}$	pen^{e}	date^{e}	chili pepper	criminology	croquet
jellyfish$_{f,e}$	grasshopper	desk	photograph	eggplant	cloves	dentistry	crosswords
lobster	hamster	dishwasher	pitchfork	ginger	dandelion	economics	darts
oyster	head lice^{f,e}	door mat$_{f}$	rake	mint	garlic	geography	fishing
plankton$_{m,f,e}$	leech	lamp$_{f,e}$	scalpel	mushroom	lettuce	geometry	frisbee
salmon	lizard	painting	scissors^{m,f,e}	olive^{m,e}	milk	literature	hiking
sardine	maggot$_{f,e}$	piano^{m,f,e}	screw$_{m,e}$	onion	parsley^{m,f}	mathematics	hunting^{m}
sea horse^{e}	mosquito	pillow	screwdriver	orange	peanut	medicine	jogging
seal	moth	plate	sewing needle^{f,e}	pine cone	pineapple	meteorology	kite flying
shark	scorpion^{f,e}	refrigerator	shovel	pomegranate$_{m}$	potato	mineralogy	mountaineering
shrimp	silkworm	rug$_{f}$	stone$_{m}$	pumpkin	rice	nutrition	picnicking
sponge	snail	shelf	string	rhubarb	sage	palm reading	skiing
squid^{m,f,e}	spider^{m,f,e}	suitcase	toothbrush	strawberry	seaweed	pharmacy	surfing
starfish	tapeworm	table	tractor	sugar beet	soybean$_{f,e}$	philosophy	swimming
tadpole	tarantula^{f}	telephone	trunk	tomato$_{f,e}$	spinach	psychology	tennis
trout	termite	television^{f,e}	umbrella	walnut^{m,f,e}	turnip	religious studies	weightlifting
whale	wasp	waste basket	varnish	watermelon$_{m}$	watercress	sociology	wrestling
