# Supplementary material for: Age-Related Degree and Criteria Differences in Semantic Categorization
Source: J Cogn. 2019 Jul 19;2(1):17. doi: 10.5334/joc.74 (PMC6640261; doi:10.5334/joc.74)
Supplement: Appendix B. — Monte Carlo Simulations. [file joc-2-1-74-s2.pdf]

Appendix B: Monte Carlo Simulations

In this paper, two main findings emerged with respect to age-related degree and criteria differences in semantic categorization. Across 8 categories and 3 data sets (Male, Female, Female Education Equated), the median of the posterior distribution of μ_{θ_O} - the mean threshold difference between the young and older adults - was negative 19 out of 24 times (79%). This finding indicates that older adults employ a lower threshold for category membership than young adults do (degree difference in semantic categorization). Across data sets, an average of 2.92 items per category (out of 24) were identified as functioning differently in the young and older adults. This finding indicates that there are several instances that young and older adults assess differently with respect to the categorization conditions (criteria difference in semantic categorization).

To assess how much confidence can be placed in these findings, we conducted a Monte Carlo simulation study to determine how likely both findings are to emerge in comparable data sets without a systematic age difference. To this end, we randomly assigned the participants in the Female Education Equated data set to two groups and redid the model analyses. Since there are an equal number of young and older participants in this data set, the random assignment of participants to groups is expected to do away with the age difference between groups as comparable numbers of young and older adults are expected to end up in both groups. We repeated this procedure 50 times for every category and tabulated the number of times the median of the posterior distribution of the mean threshold difference between groups was negative, as well as the number of times an item was classified as functioning differently in the two randomly composed groups (i.e., the number of times the posterior probability of indicator C_i exceeded .50).

Table B1: Number of times the mean threshold difference between groups was negative and an item was classified as functioning differently in the Monte Carlo simulation study.

Category	mean threshold difference		differently functioning item	
	negative	positive	no	yes
FISH	22	28	1200	0
INSECTS	22	28	1200	0
FURNITURE	26	24	1195	5
TOOLS	26	24	1200	0
FRUIT	27	23	1197	3
VEGETABLES	26	24	1199	1
SCIENCES	25	25	1200	0
SPORTS	23	27	1200	0
total	197	203	9591	9

Note. Number of mean threshold differences are out of 50 simulations per category, while number of differently functioning items are out of a total of 1200 per category (50 simulations x 24 items).

Table B1 summarizes the results of the Monte Carlo simulation study. It indicates that it is not likely that the paper's main findings are chance observations. When participants are assigned randomly to groups (irrespective of their age) the threshold difference between the two randomly composed groups is as often positive as it is negative, rather than predominantly negative as in our study. About half of the 50 simulations for each category (400 in total) yield a negative threshold difference, while the other half yield a positive difference (see first two columns of Table B1). This result is exactly what one would expect if there is no systematic difference between the groups.

Under these conditions hardly any items are classified as functioning differently in the two groups (see last two columns of Table B1). Across 8 x 50 simulated data sets with 24 items each, only 9 items were classified as functioning differently (out of a potential total of 8 x 50 x 24 = 9600). This

corresponds to an average of 0.02 differently functioning items per category, compared to an observed average of 2.92 items per category in our study. This result indicates that without a systematic age difference between the groups, criteria differences are much less likely to manifest than we have observed. Taken together, the results of the Monte Carlo simulation study support our claim that the degree and criteria differences we observed are the result of systematic age differences between the groups and not due to random sampling.
